# Analysis and prognostic significance of tumour immune infiltrates and immune microenvironment of m6A-related lncRNAs in patients with gastric cancer

**DOI:** 10.1186/s12920-022-01318-5

**Published:** 2022-07-25

**Authors:** Jiarong Huang, Jinxuan Song, xiangyu Li, Shuangfei Liu, Wentao Huang, Ziyi Shen, Yan Cheng, Shien Kou, Zhenguo Gao, Yunhong Tian, Jiani Hu

**Affiliations:** 1 Department of General Surgery, The Affiliated Nanchong Central Hospital of North Sichuan Medical College (University), Nanchong, 637000 Sichuan China; 2 Clinical Research Group, Grade 2017 in Department of Clinical Medicine, North Sichuan Medical College (University), Nanchong, 637000 Sichuan China; 3 Clinical Research Group, Grade 2019 in Department of Clinical Medicine, North Sichuan Medical College (University), Nanchong, 637000 Sichuan China; 4 Clinical Research Group, Grade 2018 in Department of Clinical Medicine, North Sichuan Medical College (University), Nanchong, 637000 Sichuan China; 5 Department of Radiology, Wayne State University, Detroit, MI USA

**Keywords:** m6A-related lncRNA, Immune infiltrates, Immune microenvironment, Gastric cancer

## Abstract

**Background:**

Studies have shown that long noncoding RNAs and N6-methyladenosine play important roles in gastric cancer. The purpose of this study was to determine the correlation and prognostic value of m6A-related lncRNAs and immune infiltration in gastric cancer.

**Methods:**

We downloaded the clinically related information and RNA-Seq transcriptome data of gastric cancer patients from the TCGA database. Univariate Cox regression analysis and Pearson analysis were used to screen out m6A-related lncRNAs. Consensus cluster analysis was used to divide the sample into two clusters, and LASSO analysis and Cox regression analysis were used to construct a risk scoring model.

**Results:**

A total of 25 lncRNA expression profiles were screened, and gastric cancer patients were divided into different subtypes. Cluster 2 had a better prognosis, but its stromal score, ESTIMATE score and immune score were low. Cluster 1 was rich in resting memory CD4 T cells, regulatory T cells, monocytes, and resting mast cells, and Cluster 2 was rich in activated memory CD4 T cells and follicular helper T cells. Thirteen lncRNAs were selected to construct a risk model, and the prognosis of gastric cancer patients in the high-risk group was poor. The expression of PD-L1 in tumours is significantly higher than that in normal tissues. Univariate and multivariate Cox regression analysis results showed that the overall survival rate was significantly related to stage and the risk score, which can be used as an independent prognostic factor. The results of the heatmap and scatter plot showed that clusters (*P* = 0.0045) and grade (G1–2, G3, *P* = 0.0037) were significantly related to prognosis. The relationship between the risk score and immune cell infiltration showed that memory B cells, resting dendritic cells, M0 macrophages, and M2 macrophages were positively correlated with the risk score, while resting mast cells, monocytes, activated NK cells, and follicular helper T cells were negatively correlated with the risk score.

**Conclusion:**

The results of this study indicate that m6A-related lncRNAs may play an important role in the prognosis of gastric cancer patients and the tumour immune microenvironment and may provide help for the treatment of gastric cancer patients.

## Background

In recent years, with the advancement of medical standards and the popularization of health concepts, the global morbidity and mortality of gastric cancer have declined, but it is still a major public health problem [1]. Gastric cancer is the fourth most common cancer and the second leading cause of cancer death in the world [2], with approximately 738,000 deaths every year [3]. As a common RNA modification, m6A exists in lncRNAs and microRNAs and plays an important role in regulating the splicing and translation of lncRNAs [4–6]. In recent years, many studies have shown that the tumour microenvironment plays an important role in cancer progression [7–10]. Studies have shown that m6A modification is closely related to the tumour microenvironment and PD-L1 expression in hepatocellular carcinoma and cholangiocarcinoma [11–14], and prognostic models of m6A-related lncRNAs can be used to predict the overall survival of patients with various tumours [15–20], but the clinical application and immunotherapy effect of m6A-related lncRNAs in the prognosis of gastric cancer are still unclear.

This study aims to evaluate the correlation of m6A-related lncRNAs with immune cell infiltration and the prognosis of gastric cancer patients. By constructing a gastric cancer prognostic model, the patients were divided into high-risk and low-risk groups to determine whether m6A-related lncRNAs could be used as prognostic biomarkers for gastric cancer.

## Materials and methods

### Data collection

The clinically related information and lncRNA expression data of gastric cancer patients were downloaded from the TCGA database. The clinically related information mainly included sex, age, classification, grade, and TNM staging. Continuous variables were converted to categorical variables, and the chi-square test was used to compare the variables in the training group and validation group.

### Identification of m6A-related genes and prognosis-related m6A lncRNAs

We extracted the expression matrix of m6A-related genes based on the mRNA expression data in the TCGA database, used the “Limma”R software package to filter m6A-related lncRNAs (*P* < 0.05, Cor > 0.5), and then visualized them as a coexpression network graph. Using univariate Cox regression analysis with *P* < 0.05 as the screening standard, prognostic m6A-related lncRNAs were screened out. The Wilcoxon statistical method was used to detect the differential expression of lncRNAs between gastric cancer tissues and normal tissues.

### Consensus clustering identifies m6A-related lncRNA subgroups

We used the “ConsensusClusterPlus” software package to cluster gastric cancer patients into different subtypes to explore the biological characteristics of m6A-related lncRNAs. Kaplan–Meier survival analysis and the log-rank test were used to analyse the differences in clinicopathological factors between the two groups.

The correlation between different clusters and the TIME was also explored.

The “ESTIMATE” software package was used to calculate the stromal, ESTIMATE and immune scores, and the Wilcoxon test was used to test the differential expression of stromal, ESTIMATE and immune scores between the two clusters. The CIBERSORT algorithm was used to evaluate the immune cell type score of each sample. The Wilcoxon test was used to show the abundance of immune infiltrating cells between the two clusters.

### Construction and validation of the risk model and its relationship with clinicopathological characteristics and immune cell infiltration

**Fig. 1:**
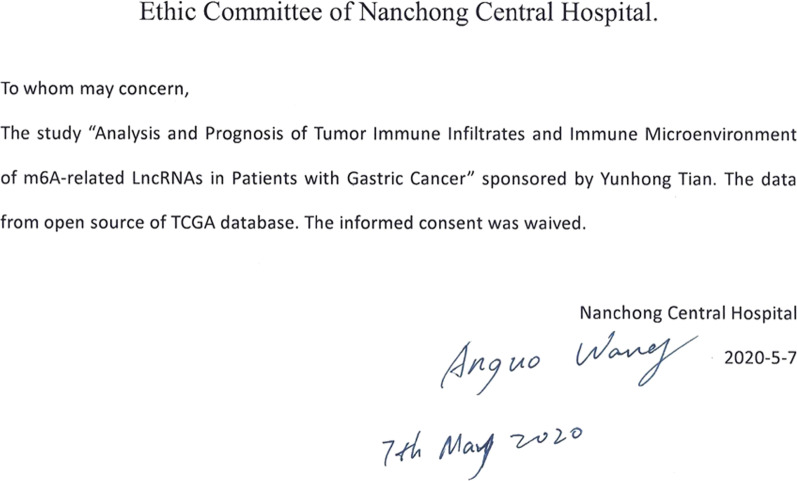
Ethic committee approval for this study

The least absolute shrinkage and selection operator (LASSO) regression algorithm was used to screen the m6A-related lncRNAs that are most closely related to overall survival. Then, a risk model was built. The formula for the risk score was coef1* × 1 + coef2* × 2 + coef3* × 3 + … + coefi*xi (where coef refers to the coefficient of each lncRNA, and X refers to the expression level of the lncRNA). According to the median risk score, the patients were divided into high-risk and low-risk groups. Kaplan–Meier survival analysis was used to detect the difference in overall survival between the high- and low-risk groups. According to clinicopathological characteristics, subgroup analysis, univariate Cox regression analysis and multivariate Cox regression analysis were performed to evaluate whether the risk score can be used as an independent prognostic factor. Pearson correlation tests were used to evaluate the relationship between the risk score and immune cell infiltration. This study was approved by the Ethic Committee of Nanchong Central Hospital (Fig. 1).

## Results

### Identification of m6A-related lncRNAs in gastric cancer (GC) patients

**Fig. 2:**
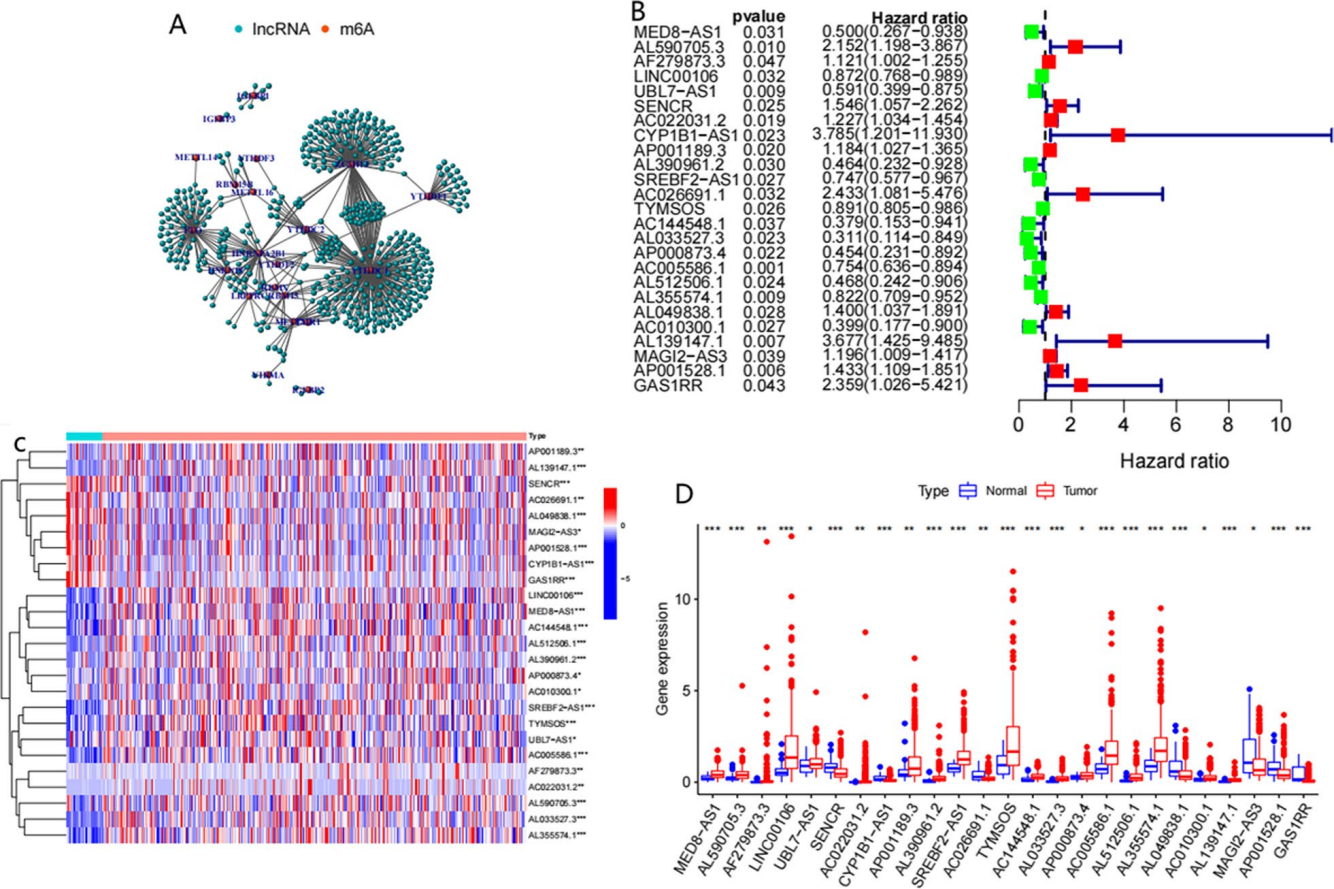
lncRNAs and m6A-related genes are significantly related **A** m6A-related genes and lncRNA coexpression network. **B **Forest plot of the prognostic ability of the 25 m6A-related lncRNAs **C** Heatmap of the 25 m6A-related lncRNAs in normal tissues and tumour tissues **D** Expression value of the 25 m6A-related lncRNAs in normal tissues and tumor tissues

We extracted the expression of lncRNAs and m6A-related genes from the TCGA database and used Pearson’s correlation value > 0.5 and *P* < 0.01 as the criteria for screening m6A-related lncRNAs. The coexpression network of m6A-related lncRNAs is shown in Fig. 2A. Through univariate Cox regression analysis, 25 lncRNAs were obtained (*P* < 0.05). The forest plot results showed that 12 lncRNAs were risk factors (HR > 1), and the remaining 13 lncRNAs were protective factors (HR < 1) (Fig. 2B). The heatmap and box plot show the expression of the 25 lncRNAs in gastric cancer tissues and normal tissues (Fig. 2C, D).

### m6A-related lncRNA consensus clustering

**Fig. 3:**
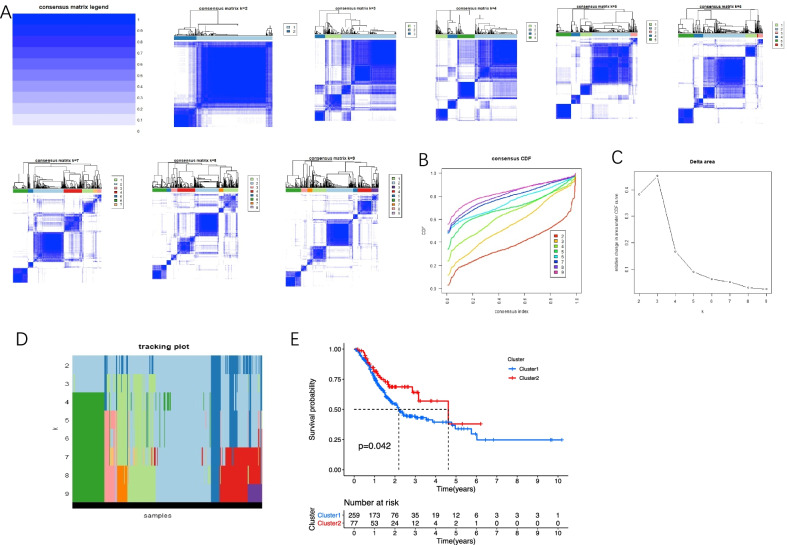
Differential survival outcome of GC in Cluster 1/2 subtypes. **A** Consensus score matrix of all samples when K = 2, K = 3, K = 4, K = 5, K = 6, K = 7, K = 8, and K = 9. **B** The cumulative distribution functions (CDFs) for K = 2–9. **C** Relative change in area under CDF area for k = 2–9. **D** Tracking plot. **E** Survival analysis of Cluster 1/2 subtypes in the TCGA cohort

Consensus clustering was used to cluster the samples, and the CDF curve showed that when k = 2, the interference between the subgroups was the smallest, and the difference was significant (Fig. 3A, B). According to the relative change in the area under the CDF curve and the tracking plot, the k value was determined to be 2 to 9 (Fig. 3C, D). The survival chart results showed that patients in the Cluster 2 subgroup had a higher overall survival rate (Fig. 3E, *P* < 0.05).

### Immune cell infiltration and tumour immune microenvironment (TIME) and genetic correlation analysis

**Fig. 4:**
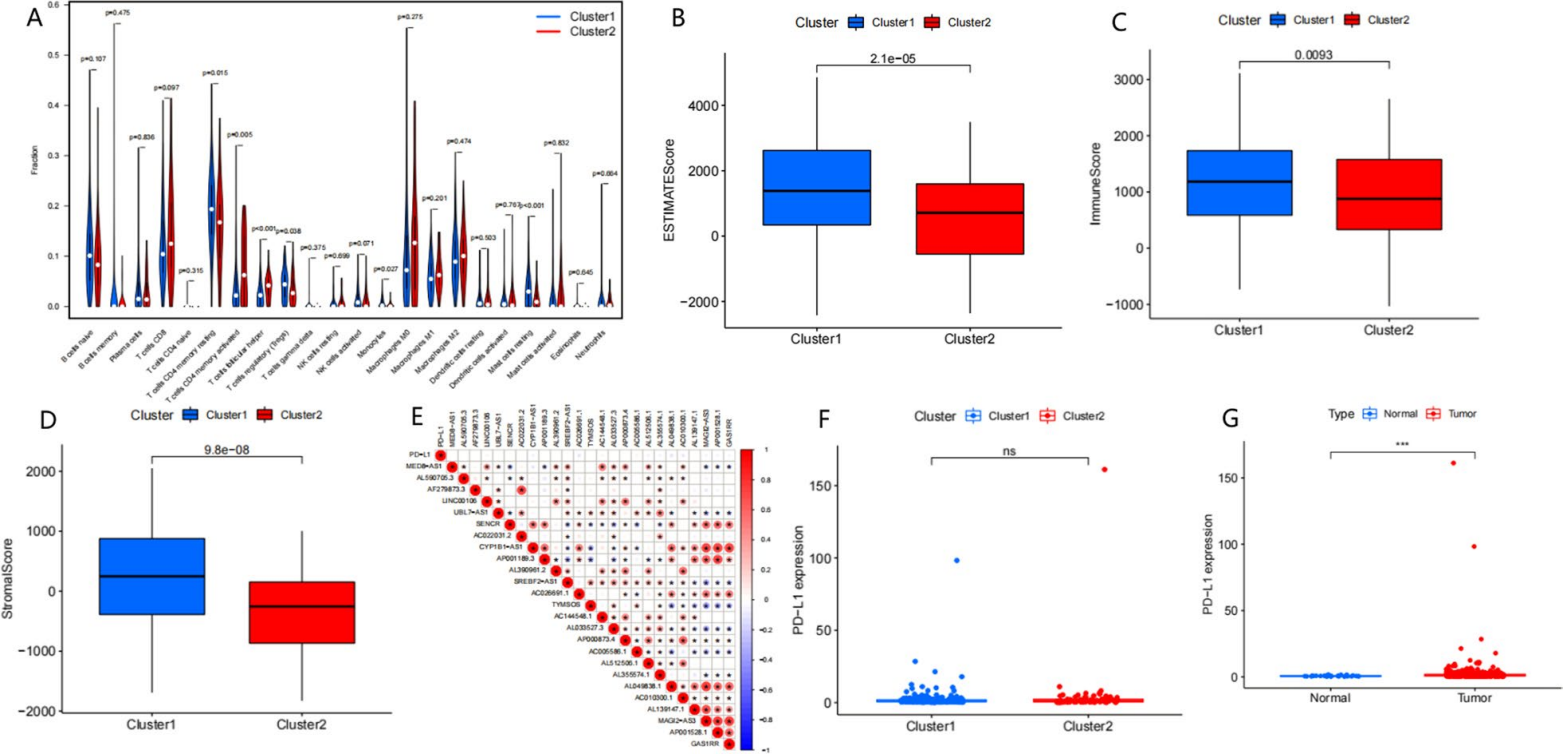
**A** The infiltrating levels of immune cell types in Cluster 1/2 subtypes. **B** ESTIMATE score in two subtypes. **C** Immune score in two subtypes. **D** Stromal score in two subtypes. **E** The correlation between m6A-related lncRNAs and PD-L1 and other genes. **F** PD-L1 expression in two clusters. **G** PD-L1 expression in tumor and normal tissues

We analysed the scores of 22 immune cell types, and the results showed that Cluster 1 was rich in resting memory CD4 T cells, regulatory T cells, monocytes, and resting mast cells (all *P* < 0.05). Cluster 2 was rich in activated memory CD4 T cells and follicular helper T cells (Fig. 4A, all *P* < 0.05). To further understand the relationship between m6A-related lncRNAs and immunity, the ESTIMATE algorithm was used to calculate the distribution difference of the immunity ratio. Cluster 1 had a higher ESTIMATE score, immune score and stromal score (Fig. 4B–D). The correlation between m6A-related lncRNAs showed that there was a positive correlation between these lncRNAs (Fig. 4E). The expression of PD-L1 in the two clusters was not significantly different (Fig. 4F). The expression of PD-L1 in tumour tissues was significantly higher than that in normal tissues (Fig. 4G).

### Construction and verification of a prognostic model based on m6A-related lncRNAs

**Fig. 5:**
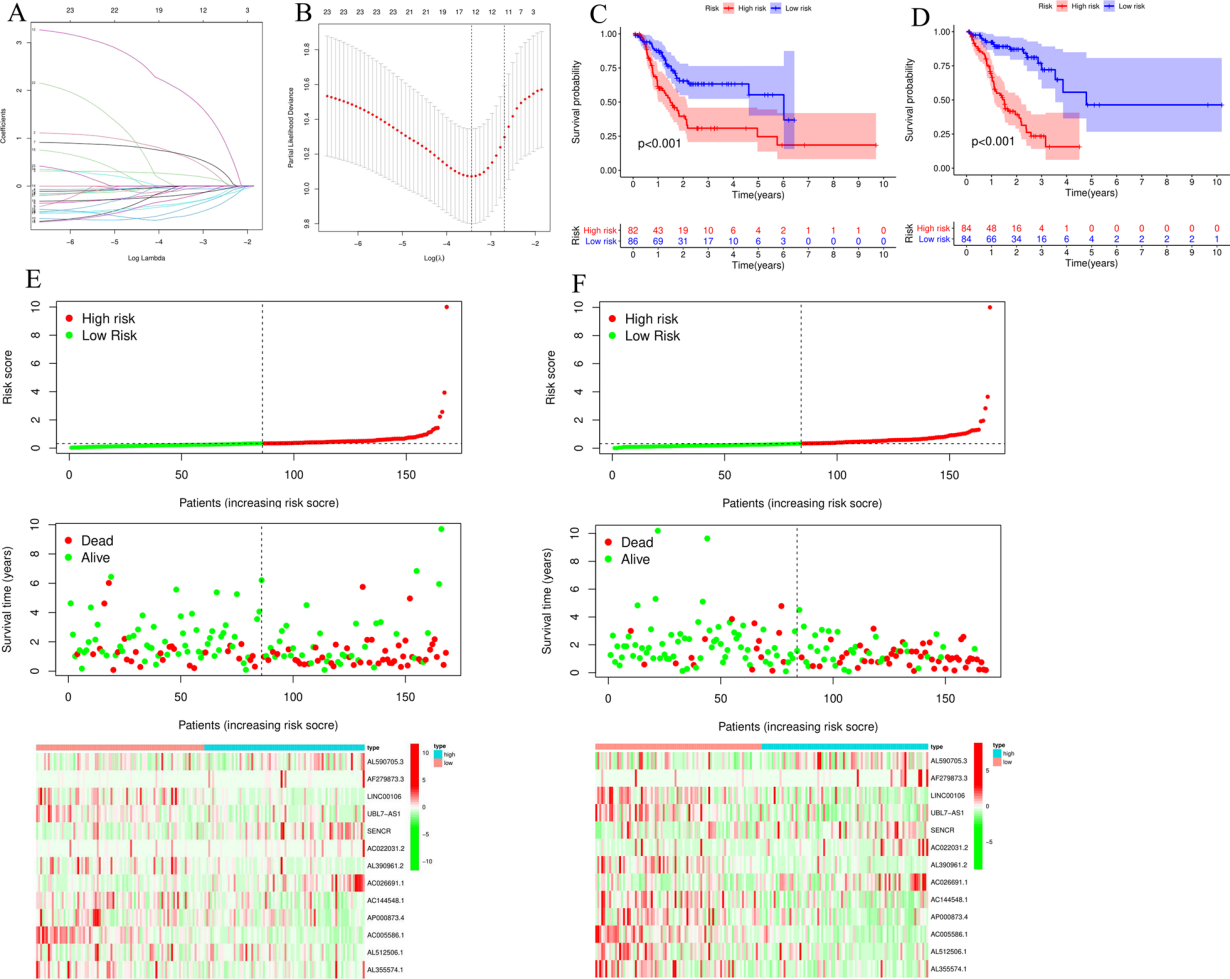
Construction of a gastric cancer prognostic risk model based on 13 m6A-related lncRNAs. **A**, **B** Least absolute shrinkage and LASSO regression. **C** Overall survival analysis of high/low-risk patients in the validation group. **D** Overall survival analysis of high/low-risk patients in the training group. **E** Distribution of risk score, survival status and heatmap of the 13 m6A-related lncRNAs in the high/low risk group in the train group. **F** Distribution of risk score, survival status and heatmap of the 13 m6A-related lncRNAs in the high/low risk group in the validation group

We used LASSO regression analysis to identify prognosis-related m6A lncRNAs (Fig. 5A, B). Based on the median risk score, we divided the gastric cancer patients into high-risk and low-risk groups. The survival analysis results showed that the survival rate of gastric cancer patients in the high-risk group was poor (Fig. 5C, D). The risk score and survival status distribution results of each patient in the training group and the validation group showed that the higher the risk score was, the higher the patient’s mortality rate and the more significantly reduced the survival time was. The heatmap showed that the expression patterns of 13 lncRNAs in the high- and low-risk groups were different (Fig. 5E, F). The above results indicate that the risk score based on 13 m6A-related lncRNAs has high predictive power for the prognosis of gastric cancer patients.

### The prognostic risk score is related to clinicopathological characteristics and immune cell infiltration

**Fig. 6:**
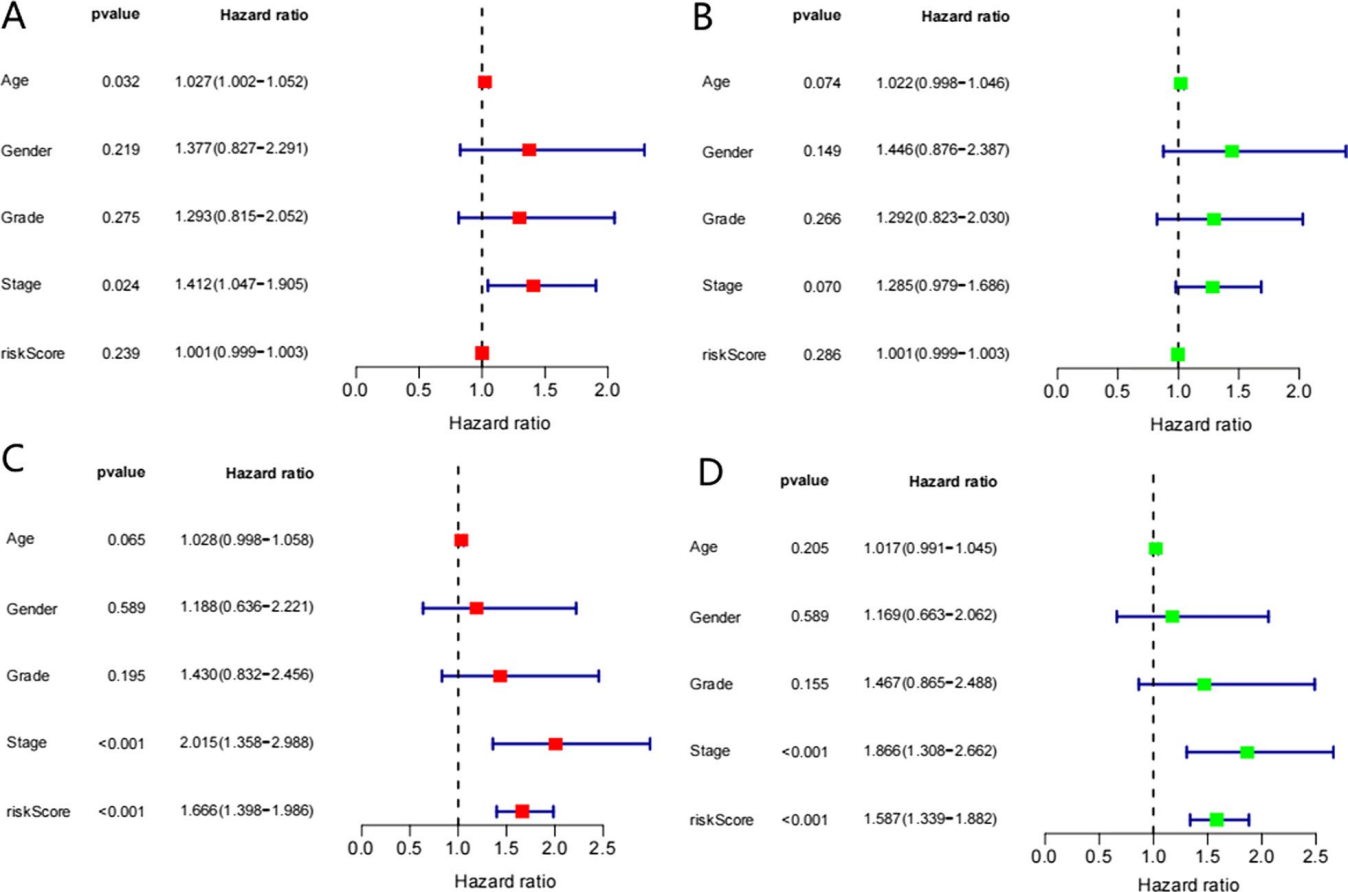
**A** Multiple Cox regression in the validation group. **B** Univariate Cox regression in the validation group. **C** Multiple Cox regression in the train group. **D** Univariate Cox regression in the train group

We used univariate and multivariate Cox regression analyses to determine whether the risk model based on m6A-related lncRNAs can be used as an independent prognostic factor for predicting gastric cancer patient survival. Univariate Cox regression analysis showed that the overall survival rate was significantly related to stage and risk score. Stage (HR = 1.866, *P* < 0.001) and risk score (HR = 1.587, *P* < 0.001) were independent prognostic factors in the training group. The results of multivariate Cox regression analysis showed that age (HR = 1.027, *P* < 0.032) and stage (HR = 1.412, *P* < 0.024) were independent prognostic factors in the validation group. Stage (HR = 2.015, *P* < 0.001) and risk score (HR = 1.666, *P* < 0.001) were independent prognostic factors in the training group (Fig. 6A–D).

**Fig. 7:**
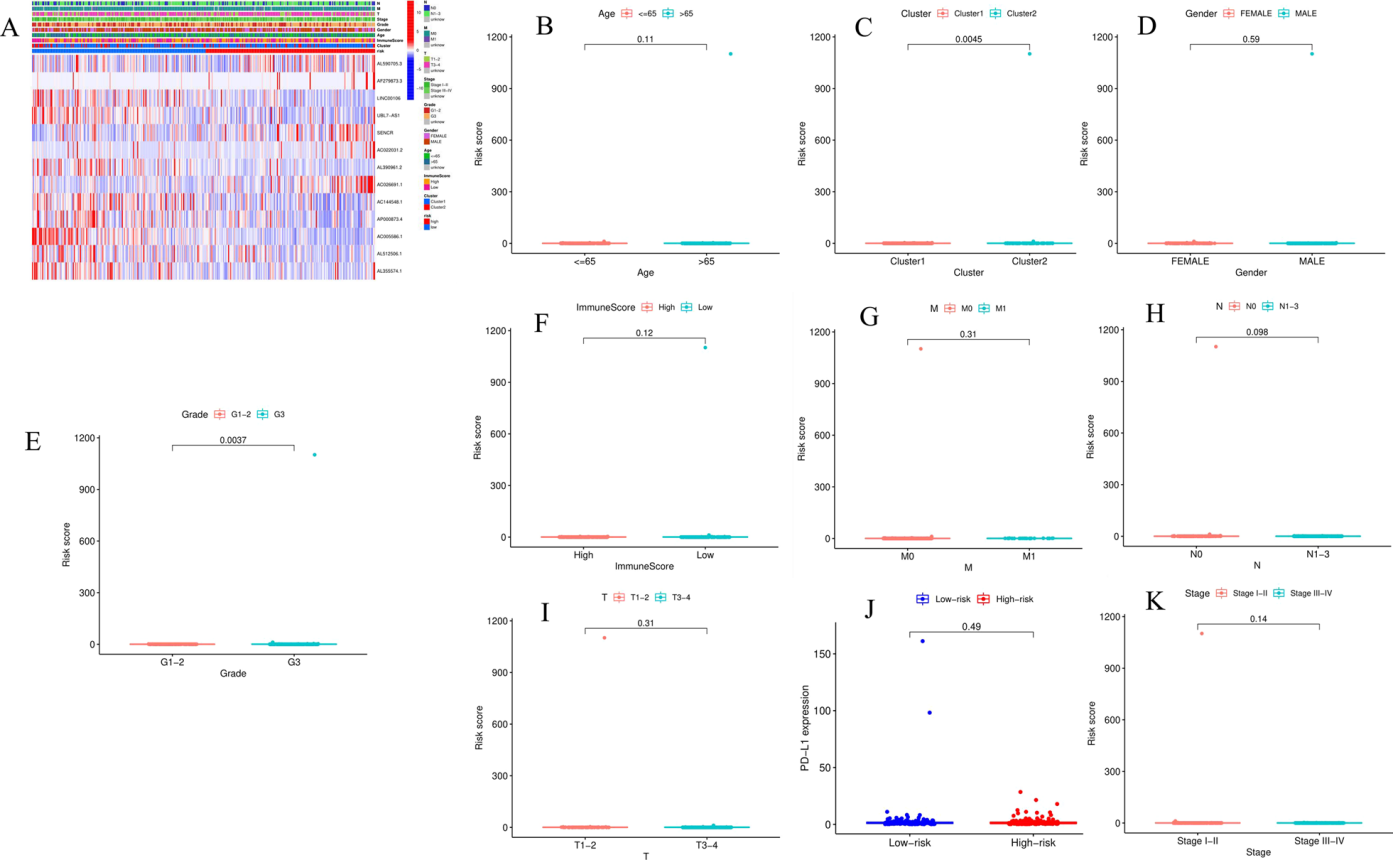
The prognostic risk score is related to clinicopathological characteristics. **A** Heat maps of clinicopathological characteristics, immune scores and different lncRNA expression patterns in the high and low-risk groups. **B** Distribution of risk scores stratified by age. **C** Distribution of risk scores stratified by Cluster 1/2. **D** Distribution of risk scores stratified by sex. **E** Distribution of risk scores stratified by grade. **F** Distribution of risk scores stratified by immune score. **G** Distribution of risk scores stratified by M stage. **H** Distribution of risk scores stratified by N stage. **I** Distribution of risk scores stratified by stage. **J** Distribution of risk scores stratified by T stage. **K** PD -L1 expression in high/low risk

**Fig. 8:**
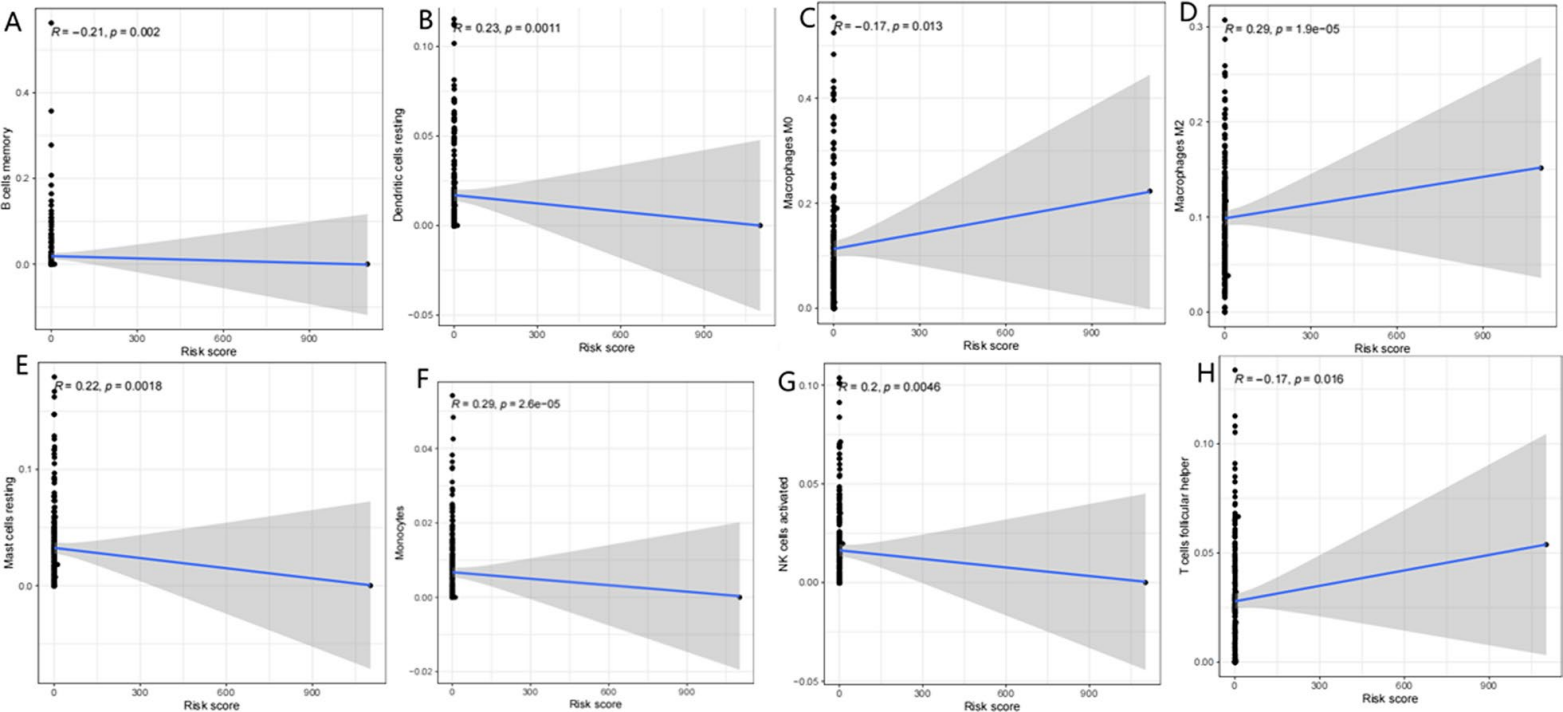
Relationship between the risk score and infiltration level of eight immune cell types. **A** B cells memory. **B **Dendritic cells resting. **C** Macrophage M0. **D** Macrophages M2. **E **Mast cells resting. **F **Monocytes. **G** NK cells activated. **H** T cells follicular helper

We further evaluated the relationship between the risk score and prognosis of 13 m6A-related lncRNAs. The results of the heatmap and scatter plot showed that clusters (*P* = 0.0045) and grade (G1-2, G3, *P* = 0.0037) were significantly related to prognosis (Fig. 7A–K). We further explored the relationship between the risk score and immune cell infiltration (Fig. 8A–H), and the results showed that memory B cells, resting dendritic cells, M0 macrophages, and M2 macrophages were positively correlated with the risk score (all *P* < 0.01), while resting mast cells, monocytes, activated NK cells, and follicular helper T cells were negatively correlated with the risk score (all *P* < 0.01).

## Discussion

Characterized by its low early diagnosis rate, high malignancy and poor prognosis, GC is a serious threat to human health in our country. At present, the process of the malignant progression of GC has not been fully elucidated. The identification of new biomarkers for the diagnosis and prognosis of GC has become a prerequisite to successfully treat GC. Accumulating evidence indicates the significant influences of lncRNAs on tumorigenesis [21, 22]. LncRNAs impact various dimensions in the occurrence and progression of tumours, especially at the transcriptional and posttranscriptional levels [23]. For example, Ning Cui experimentally observed [24] that LINC00511 acted as a therapeutic target in GC treatment and could regulate the expression of STAT3 via miR-625-5p. LINC00649 functions as an oncogenic lncRNA by accelerating cell proliferation, migration and epithelial–mesenchymal transition [25]. With the continuous development of molecular biotechnology, lncRNAs related to GC have been discovered, but our understanding of them is still in rudimentary stages. As the most prevalent internal modification of RNA, m6A has been thoroughly and widely studied recently. Published reports indicate that m6A-related lncRNAs could affect both the occurrence and progression of GC. Four m6A-methylated and expressed lncRNAs were identified, including RASAL2-AS1, LINC00910, SNHG7 and LINC01105, which exert regulatory roles on GC cell proliferation [26]. However, the prognostic significance of m6A-related lncRNAs needs to be further explored.

This study analysed m6A-related lncRNAs related to the clinical characteristics of gastric cancer patients and found that 12 lncRNAs were risk factors (HR > 1) and 13 lncRNAs were protective factors (HR < 1). The risk score constructed from 13 m6A-related lncRNAs was significantly related to overall survival, clusters and grade (G1–2, G3). Cluster 1 was related to the high-risk group, and its prognosis was poor.

M6A regulators, such as writers, readers and erasers, play important roles in cancer. Our results showed that 12 lncRNAs were risk factors (HR > 1), and 13 lncRNAs were protective factors (HR < 1) for gastric cancer. We constructed a risk score model based on thirteen m6A-related lncRNAs, including LINC00106, TYMSOS, MED8-AS1, SREBF2-AS1, AL390961.2, AC144546.1, and AC005586.1. Our results show that the risk model based on m6A-related lncRNAs is reliable. Wang and his colleagues found that the expression of LINC00106 in thyroid cancer was significantly lower than that in normal tissues. LINC00106 suppresses the metastasis and invasion of cancer cells by inhibiting epigenetic-mesogenic transition as a tumour suppressor [27]. A risk score model was developed based on five lncRNAs: LINC00205, TRHDE-AS1, OVAAL, LINC00106, and MIR100HG. Wu verified that this [28] lncRNA-based risk model performs well in predicting GC prognosis. LINC00106 could be regarded as a significant prognostic biomarker in gastric cancer [29], which was in agreement with the findings of our study. Studies have found [30] that TYMSOS is overexpressed in GC cells and exerts growth-promoting effects on GC. Moreover, TYMSOS, as a competitive endogenous RNA, modulates GC progression at the posttranscriptional level. Unlike the above mentioned investigations, the lncRNAs AL390961.2, AC144546.1, and AC005586.1 were first reported in GC, leaving a wide scope for further research.

A number of studies in recent years have shown that m6A can play an important role in cancer immunity through different regulatory factors [31–34]. The m6A regulatory factor in colon cancer has three m6A modification modes, and these three m6A modification modes are closely related to the immunophenotype [35]. The tumour immune microenvironment plays an important role in immunotherapy. It has been reported that tumour-infiltrating lymphocytes in the tumour microenvironment promote disease progression and increase the chance of invasion and metastasis. M2 macrophage infiltration is correlated with a poor prognosis in colon cancer [36]. Furthermore, a higher fraction of M0 macrophages (*P* = 0.001) and a lower fraction of M2 macrophages (*P* = 0.018) were found to be risk factors for a poorer histological grade of hepatocellular carcinoma [37]. Some studies have reported that tumour-associated macrophage infiltration is associated with invasion, angiogenesis, and poor prognosis in GC [38]. This study found that Cluster 2 was rich in activated memory CD4 T cells and follicular helper T cells and has low activation in stromal cells. The results of the relationship between the risk score and immune cell infiltration showed that memory B cells, resting dendritic cells, M0 macrophages, and M2 macrophages were positively correlated with the risk score (all *P* < 0.01), while resting mast cells, monocytes, activated NK cells, and follicular helper T cells were negatively correlated with the risk score (both *P* < 0.01). In summary, we analysed stromal cells and infiltrating immune cells in the tumour immune microenvironment, and the results showed that m6A-related lncRNAs were involved in the reprogramming of the tumour immune microenvironment.

This study still has some limitations. First, the effectiveness of this model still needs to be verified in a large number of external samples. Second, the m6A-related lncRNAs selected in this study need to be verified by further functional experiments. It is necessary to further reveal the regulatory network between m6A and lncRNAs.

In conclusion, this study screened 25 m6A-related lncRNAs. Different gastric cancer patients have different lncRNA subtypes in terms of overall survival, and the overall survival of Cluster 2 is higher. The prognostic risk score of m6A-related lncRNAs is closely related to clinicopathological characteristics (grade), the two subtypes and immune cell infiltration.

## Data Availability

The datasets generated and analyzed during the current study are available in the TCGA repository, [https://www.cancer.gov/about-nci/organization/ccg/research/structural-genomics/tcga].

## Abbreviations

GC: Gastric cancer
CDFs: Cumulative distribution functions
TIME: Tumor immune microenvironment
TCGA: The cancer genome atlas
